# An Inexpensive Biomechanical Model to Help Teach and Learn Newer Mandible Reduction Techniques

**DOI:** 10.5811/cpcem.1416

**Published:** 2023-05-30

**Authors:** Shantal Tummings, Sierra Garrett, Ja’Neil G. Humphrey, Jasmine Haddad, Jon Roper, Deena I. Bengiamin, Timothy P. Young

**Affiliations:** * Loma Linda University School of Medicine, Laboratory for Innovations in Medical Education, Loma Linda, California; † Loma Linda University Medical Center and Children’s Hospital, Department of Emergency Medicine, Loma Linda, California

Dear Editor:

While preparing for a mandible reduction teaching session, we were happy to find Lum and Poh’s description of the successful use of the wrist pivot method for a refractory dislocation.^1^ Like the authors, we have encountered difficulty with the conventional method of mandible dislocation reduction. In this technique, the clinician places both thumbs in the patient’s mouth along the lower molars and applies downward, backward force. Simultaneous reduction of both sides of a bilateral dislocation requires considerable force on the part of the clinician, which is difficult for the patient to tolerate. Even when successful, the conventional approach places the proceduralist’s thumbs at risk of injury from masseter spasm; they must be protected by gauze wraps or a bite block.

In the last two decades, two techniques have been proposed as alternatives to the conventional approach: the wrist pivot and extraoral methods.^2^,^3^ The authors used the wrist pivot method, which uses different hand positioning and is better tolerated than the conventional method but still requires the clinician’s fingers in the patient’s mouth.^4^ The extraoral technique allows the proceduralist to avoid placing fingers in the patient’s mouth, but it is more difficult for physicians to perform.^4^ However, these techniques are difficult to understand and learn from static images.

While the authors found success with the wrist pivot method after eight doctors attempted to use the conventional method with and without sedation, Image 1 of the authors’ article shows hand positioning that is different from the original description of the method (https://bit.ly/3Tf5RRO).^2^ This is not surprising, considering that they employed the technique following written instructions only. They also illustrate and discuss the extraoral method. Like the authors’ experience with the wrist pivot method, we initially misread the original description of this technique and taught and used a slightly different method. In our experience, hands-on practice with a trainer is the best way to learn complicated techniques in the way that authors intended them to be performed. This can be aided by video demonstrations, which can help clarify hand positioning and force application. However, we could not locate any commercial mandible reduction trainers. To remedy this, we created an inexpensive, biomechanical model.

We bought a plastic human skull model (www.amzn.com/B07R9NYPCN), removed the temporomandibular joint (TMJ) capsules, and drilled 1/8″ holes at the center of the TMJ and 7/64″ holes through the top of the condyles (Image 1). We looped elastic string (www.amzn.com/B088CQR4PL) through the TMJ and condyles and secured it with toggle stoppers (www.amzn.com/B08M7Z2XY1). Resistance bands (www.amzn.com/B01AVDVHTI) simulate masseter muscles, looped around each zygomatic arch, tied at the base of the mandible, and held in place by a drywall screw on each side. Since the skull model that we bought had a removable cranium cap, we used a nut and fender washer to hold it in place. The total cost for parts was $67. A video explaining how the trainer works can be viewed here: youtu.be/OLPu0vwmTpo.

During our practice session, we had an assistant hold the trainer on a gurney with the head of the bed elevated (Image 2). This is the same positioning approach we use for real patients. For the conventional method, we were able to demonstrate and practice a sequential approach, in which each side is reduced in succession. This approach may be successful when a bilateral simultaneous approach fails.^5^ While practicing the extraoral technique, it became clear that the application of force in opposite directions on either side of the mandible is key (Image 3). This causes a rotational motion like the one employed in a conventional sequential reduction. The motion of the wrist pivot method disengages the condyle from the skull base and allows it to clear the articular eminence located anterior to the mandibular fossa. We were able to directly visualize this with the trainer. The trainer’s utility is best conveyed by moving images; so we made a video that can be viewed (Video).

We agree with the authors’ statement that familiarity with multiple techniques is important for refractory mandible dislocations. We add that learning proper technique using trainers increases chances of success.

This project was a product of the Laboratory for Innovations in Medical Education, which is supported by a grant from the Kiwanis Cal-Nev-Ha Children’s Fund.

## Supplementary Information

Video Trainer explanation of mandible reduction

## Figures and Tables

**Image 1:**
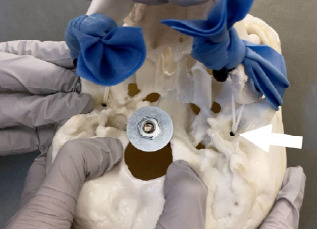
We drilled a hole in the center of the articular fossa (arrow) and one through the condyle, and then secured the condyle with elastic string.

**Image 2:**
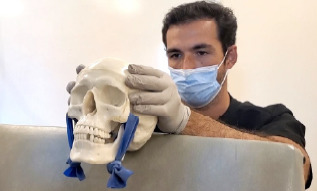
The completed mandible reduction trainer.

**Image 3:**
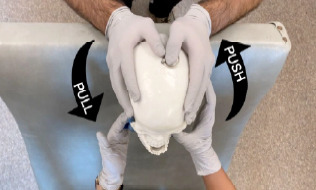
The extraoral reduction technique. On one side, the thumb pushes on the coronoid process while the other hand grasps the angle of the mandible and pulls. The process is repeated on the opposite side for bilateral dislocations.
